# Assessment of Vascular Function in Response to High-Fat and Low-Fat Ground Beef Consumption in Men

**DOI:** 10.3390/nu15061410

**Published:** 2023-03-15

**Authors:** Jason R. Lytle, Sean T. Stanelle, Steven E. Martin, Stephen B. Smith, Dana R. Smith, Stephen F. Crouse

**Affiliations:** 1 KBR, Houston, TX 77058, USA; 2 Department of Kinesiology and Sport Management, Texas A&M University, College Station, TX 77843, USA; 3 Department of Animal Science, Texas A&M University, College Station, TX 77843, USA; 4 Independent Nutrition Consultant, College Station, TX 77845, USA

**Keywords:** vascular function, flow-mediated dilation, carotid–femoral pulse wave velocity, cardiovascular disease risk, beef, diet, cholesterol, blood pressure

## Abstract

Red meat is stigmatized as an unhealthy protein choice; however, its impacts on vascular function have not been evaluated. We aimed to measure the vascular impact of adding either low-fat (~5% fat) ground beef (LFB) or high-fat (~25% fat) ground beef (HFB) to a habitual diet in free-living men. Twenty-three males (39.9 ± 10.8 years, 177.5 ± 6.7 cm, 97.3 ± 25.0 kg) participated in this double-blind crossover study. Assessment of vascular function and aerobic capacity were measured at entry and in the last week of each intervention and washout period. Participants then completed two 5-week dietary interventions (LFB or HFB; 5 patties/week) in a randomized order with a 4-week washout. Data were analyzed via 2 × 2 repeated-measures ANOVA (*p* < 0.05). The HFB intervention improved FMD relative to all other time points, while lowering systolic (SBP) and diastolic blood pressure (DBP) relative to entry. Neither the HFB nor the LFB altered pulse wave velocity. The addition of either low- or high-fat ground beef did not negatively alter vascular function. In fact, consuming HFB improved FMD and BP values, which may be mediated by lowering LDL-C concentrations.

## 1. Introduction

Cardiovascular disease (CVD) is the leading causes of mortality globally [1]. Physiological factors that increase CVD risk include central obesity, elevated blood pressure (BP), and dyslipidemia [2]. Vascular function is closely related to CVD risk, and measures of vascular function are accepted independent risk markers for CVD. Red meat consumption has been associated with CVD risk and mortality due to its relatively large content of saturated fatty acids (SFAs), cholesterol, and heme iron (i.e., ferritin) [3,4,5,6,7]. Nevertheless, the greatest risk is often seen with processed meat consumption and in at-risk populations, with little or no association observed for unprocessed meat consumption or plasma ferritin concentrations when red meat is consumed in recommended doses [4,5,6,7,8,9,10]. The association is further called into question by studies that observed minimal reduction in CVD risk and mortality in dietary patterns with less red meat intake [7,11,12]. To the authors knowledge, the direct impact of red meat on vascular function has yet to be evaluated.

Vascular endothelial damage and dysfunction are the initiating steps of atherosclerosis. Endothelial dysfunction is identified by an impaired vascular response to dilators, most commonly associated with decreased nitric oxide (NO) bioavailability [13,14]. Brachial artery flow-mediated dilation (FMD) is a non-invasive measure of NO bioavailability that assesses endothelial function by measuring the vasodilatory response to reactive hyperemia [14,15,16]. FMD cutoff values of 6.5% and 7.1% for ‘optimal’ endothelial function have been proposed as clinical standards for European and Japanese individuals, respectively, with values lower than this suggesting increased CVD risk [17,18]. Additionally, vascular function is commonly assessed by the carotid–femoral pulse wave velocity (PWV), an indicator of arterial elasticity [19,20]. PWV increases with arterial stiffness and is associated with higher rates of cardiovascular events [21]. Together, FMD and PWV comprehensively assess vascular health and CVD risk in humans [14,19,20].

Few studies have evaluated the chronic dietary influence on vascular function. De Roos et al. [22,23] reported that diets high in *trans*-fatty acids (TFAs), which lower high-density lipoprotein cholesterol (HDL-C) concentrations, reduced the FMD response by 1.8% relative to a high-SFA diet (41% total fat, <1% TFAs) [23]. No difference was observed between a low-fat, high-carbohydrate (CHO) diet (60% energy from CHO, 25% energy from fat (7.8% energy from monounsaturated fatty acids [MUFAs]) and an oil-rich diet high in MUFAs (38% energy as CHO, 44% energy as fat (19% energy from MUFAs)) [22]. Comparing diets high in either MUFAs or SFAs, the SFA diet resulted in the lowest FMD whereas the MUFA diet elevated the FMD response [22]. The two studies that assessed PWV demonstrated no significant effect of dietary intervention [24,25]. 

Endothelial dysfunction often occurs along with elevated BP, with an inverse relationship observed between BP and vascular function as assessed by FMD [26]. Endothelial function is markedly attenuated in individuals with elevated BP [27], while chronically elevated BP itself can damage the endothelium and promote the atherogenic process [28]. Replacing CHO with either MUFAs or SFAs in healthy populations does not negatively affect BP [24,29]. Further, an isocaloric intervention consisting of either high SFAs or MUFAs resulted in lower systolic BP (SBP) and diastolic BP (DBP) following the MUFA diet, with no change following the SFA diet [30]. These results indicate that isocaloric diets high in total fat do not increase BP, while high-fat diets enriched with MUFAs can reduce BP. 

The purpose of this study was to assess the vascular impact, measured via FMD, PWV, and BP, of 5 weeks of high-fat ground beef (HFB) or low-fat ground beef (LFB) consumption in men. Our central hypothesis was that there would be no significant difference in FMD and PWV measures following the beef interventions, but that the added MUFAs of the HFB intervention would lower BP relative to the LFB intervention. Exploratory analyses were completed on dietary outcomes, aerobic fitness, and energy expenditure.

## 2. Materials and Methods

### 2.1. Participant Recruitment

This randomized, controlled, two-period, crossover trial was conducted in accordance with the Declaration of Helsinki guidelines [8]. This trial was registered at www.clinicaltrials.gov as NCT04841460 (accessed on 12 April 2021). All procedures involving human participants were approved by the Texas A&M University Institutional Review Board for use of human participants in research (protocol number IRB2018-0755). Participants were recruited from the Bryan/College Station area in October and November 2019, and the ground beef treatments were initiated in February 2020. The final blood samples were collected in July 2020. Study staff were not blinded, but the statistician was blinded to treatment during the initial analyses by identifying the diet conditions as A and B. All participants were provided detailed instructions, including the potential risks of participation.

The data presented in this paper were collected on a subsample of the men who were sampled as part of a larger study in Lytle et al. [31], which provides an in-depth description of participant recruitment, including inclusion and exclusion criteria. Briefly, participants must not have been consuming restrictive diets or cholesterol-lowering medications and needed to be normocholesterolemic (120–300 mg/dL). Participants were advised not to change their habitual diet or level of physical activity. This was assessed by 3-day food logs, and by 7-day activity logs and submaximal VO_2_ treadmill tests completed at entry and after all interventions, respectively. Of the 32 men who were randomly assigned to treatment groups (LFB or HFB), 7 men left the study either voluntarily or were excluded due to inability to comply (did not supply all diet records or did not provide all blood samples) and 2 men declined to provide vascular measures. A complete recruitment flow diagram is provided in Lytle et al. [31]. Twenty-three men (39.9 ± 10.8 years, 177.5 ± 6.7 cm, 97.3 ± 25.0 kg) completed all phases of the study. Participant demographics at entry to the study are listed in Table 1.

### 2.2. General Procedures

A two-period, randomized, cross-over design was used based on previous studies [32,33,34]. Two weeks prior to starting the diet intervention, participants visited the lab for an initial assessment of vascular function, body composition, and aerobic capacity. These data were used as entry time point measures. Each participant then completed two 5-week ground beef interventions (5 ground beef patties/week) in a randomly assigned order with a 4-week washout period between the test periods. The two treatments were low-fat (LFB, ~5% fat) and high-fat (HFB, ~25% fat) ground beef. Participants were assigned to one of two groups balanced with regard to plasma HDL-C concentrations measured at the initial screening (LFB first, *n* = 11; HFB first, *n* = 12). The procedures used for blood sampling and analysis have been reported in Lytle et al. [31]. Laboratory testing for vascular function and aerobic capacity were repeated in the last week of each intervention (LFB or HFB) and in the washout period.

### 2.3. Sources of Ground Beef and Food Logs

Our methods have been previously published in detail [31]. In brief, ground beef was processed by standard procedures to produce 115 g patties, individually vacuum-packaged and frozen. Chemical analysis showed the LFB and HFB patties to be 5.61% and 23.63% fat, with MUFA/SFA ratios of 1.16 and 1.05, respectively. 

Diet records from previous studies indicated that most study participants pan-broiled the ground beef patties intact, thus the samples used for this study (low- and high-fat) were pan-broiled [35] by the research team, and total fat and fatty acid composition of the cooked patties were measured [32,34,36] to determine the fat content prior to distributing the patties to the participants. Cooking losses for the LFB and HFB patties were 3 and 41%, respectively. Total fat and fatty acid per patty were calculated based on final patty weight and total lipids per patty, reported in Lytle et al. [31]. Cooked total lipid and fatty acid composition values were used by the registered dietician nutritionist (RDN) for the calculation of their contribution to the daily intake of dietary fats. As with our sources of ground beef, our dietary tracking methods have been previously published in detail [31]. Briefly, participants were required to complete a 2-week run-in period in which they documented their habitual dietary intake using the smartphone application My Fitness Pal (http://www.myfitnesspal.com, accessed on 20 February 2019) or by manual logging if a smart phone was not available. Food diaries were kept two weeks before the diet interventions and during the final two weeks of each intervention to establish nutrient intakes. The smartphone app allowed for determining whether meat sources were being replaced by the test ground beef patties or whether the patties were added to the diet. This was not available in previous studies using only dietary analysis software [32]. Previous studies by Smith and colleagues [32,36,37] indicate strong compliance to consumption of the ground beef patties themselves, with no complaints as to the flavor and texture of the patties. The phone and email communications by the RDN indicated no aversion to the taste, visual appeal, or palatability of the ground beef. This was expected, as the ground beef was formulated by standard industry procedures for the preparation of lean and high-fat ground beef.

### 2.4. Carotid–Femoral Pulse Wave Velocity

Carotid–femoral PWV measures were acquired based on previously published guidelines [38]. Participants were in a fasted state and abstained from alcohol within 24 h of their laboratory visit. Briefly, PWV measures were made via ultrasonography (Logic P6, GE Healthcare, UK, Chalfont Saint Giles) on the right carotid and femoral arteries after a 10 min supine rest. Time was measured from the top of the R wave on the QRS complex to the start of the inflection point on the pulse wave recording on six separate cardiac cycles for both the carotid and femoral artery. The average of these was used as the time measure for the PWV calculation. The actual distance between the carotid and femoral sites was measured in a straight line, and 80% of this measured distance was used in the PWV calculation. This method is demonstrated to be the most accurate means of assessing the distance between the carotid and femoral arteries in humans [38]. Finally, the difference in the averaged time delay between the carotid and femoral sites was divided by 80%, the measured distance between the sites, to produce the PWV value in meters per second. 

### 2.5. Flow-Mediated Dilation

Assessment of FMD was accomplished using a Logic P6 ultrasound machine (GE Healthcare, UK) according to previously published guidelines [39]. Briefly, after resting supine in a temperature-controlled room for 10 min following an overnight fast, participants abducted and externally rotated their right arm to increase visualization of the brachial artery. The abducted arm was placed in a padded holder atop a table level with the participant’s body to increase comfort and minimize movement during imaging. The image of the brachial artery was acquired via high-frequency linear transducer (10–12 MHz), and the baseline vessel diameter and pulse wave were recorded and saved to DVD (DVO-1000MD, Sony, New York City, NY, USA) or the ultrasound machine, respectively. After the baseline recording, a blood pressure cuff was wrapped around the participant’s forearm distal to the imaging site and inflated to 200 mmHg. The cuff was released after 5 min of occlusion, and the post-occlusion pulse wave was recorded at 15 s. Next, the post-occlusion vessel diameter was recorded from 30 to 120 s and recorded to DVD. DVD recordings were converted to MP4 files and analyzed by an individual technician via a brachial analyzer tracking software (Brachial Analyzer, Medical Imaging Applications-LLC, Coralville, IA, USA). All diameter measurements were automatically made at the end of diastole using the gating software upgrade package (Software-Gating module Add-on, Medical Imaging Applications-LLC, Coralville, IA, USA). Both gated and allometrically scaled FMD values were recorded. 

### 2.6. Body Composition

Body composition was assessed at the entry visit using a Lunar Prodigy dual-energy X-ray absorptiometry (DXA) machine (General Electric, Madison, WI, USA). The derived variables of interest were total body mass, lean body mass, fat mass, and percent body fat. Body mass index (BMI) was calculated from the measured height and weight for consideration relative to measures of lean and fat mass.

### 2.7. Submaximal Oxygen Consumption

To account for the potential confounding factor of changes in aerobic fitness altering vascular measures, oxygen uptake ($\overset{\cdot }{V}O_{2}$) was measured both before and after the ground beef interventions as an index of cardiovascular aerobic capacity [40,41]. An incremental graded exercise test to the 80% age-predicted maximal heart rate [42,43] was conducted on a motor-driven treadmill according to the Bruce protocol [43]. Oxygen consumption during the exercise was continuously measured using a calibrated metabolic gas-analysis system (Ultima^®^, Medical Graphics, Minneapolis, MN, USA). Measured $\overset{\cdot }{V}O_{2}$ was recorded as the highest 15 s average achieved during the exercise test. Estimations of participant’s maximal oxygen uptake (VO_2max_) before and after the beef interventions were calculated using an individualized linear regression based on heart rate and VO_2_ at each stage of the Bruce protocol [43]. 

### 2.8. Statistical Analysis

All data analysis was performed using IBM SPPS Statistics for Windows (Version 27.0. Armonk, NY, USA: IBM Corp). The primary statistical model was a 2-Condition (HFB, LFB) by 2-Test (entry, washout) (2 × 2) repeated-measures ANOVA when values for all four time points were available (entry, LFB, washout, HFB). Follow-up simple main effects was used for significant interactions, and a paired-samples *t*-test was used for significant condition or test effects to identify the source. If all four time points were not available (e.g., VO_2max_, energy expenditure, and DXA body composition), a paired *t*-test was used. The level of significance for all statistical measures was held at an alpha level of 0.05, and all data are reported as mean ± SD.

## 3. Results

### 3.1. Flow-Mediated Dilation

**Figure 1:**
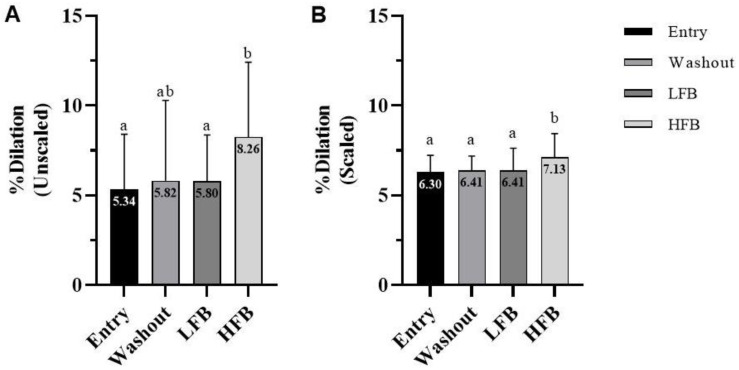
(**A**) Gated flow-mediated dilation. (**B**) Allometrically scaled flow-mediated dilation. Values are percent dilation for entry, washout, LFB intervention, and HFB intervention. Cross-over design (*n* = 23); values represent mean ± SD. Means without a common letter differ, *p* < 0.05.

Average values for gated and allometrically scaled flow-mediated dilation are depicted in Figure 1A,B as % dilation for each time point. Results for gated FMD revealed a significant main effect of test (*p* = 0.035), with the follow up paired *t*-test showing a greater FMD response after the HFB intervention compared to the entry and LFB time points (*p* = 0.008 and 0.028, respectively). Additionally, there was a trend for increased FMD response after HFB intervention compared to the washout time point (*p* = 0.057). For allometrically scaled FMD, there was also a significant main effect of test (*p* = 0.044), such that the FMD response after the HFB intervention was greater compared to the entry, washout, and LFB time points (*p* = 0.013, 0.049, and 0.028, respectively). No difference in the baseline diameter of the brachial artery and the time to peak dilation for each study time point was observed. Table 2 lists values for the baseline artery diameter, peak artery diameter, and time to peak dilation measured at each study visit. No significant condition, test, or interaction effect was observed between any study visit.

### 3.2. Pulse Wave Velocity

**Figure 2:**
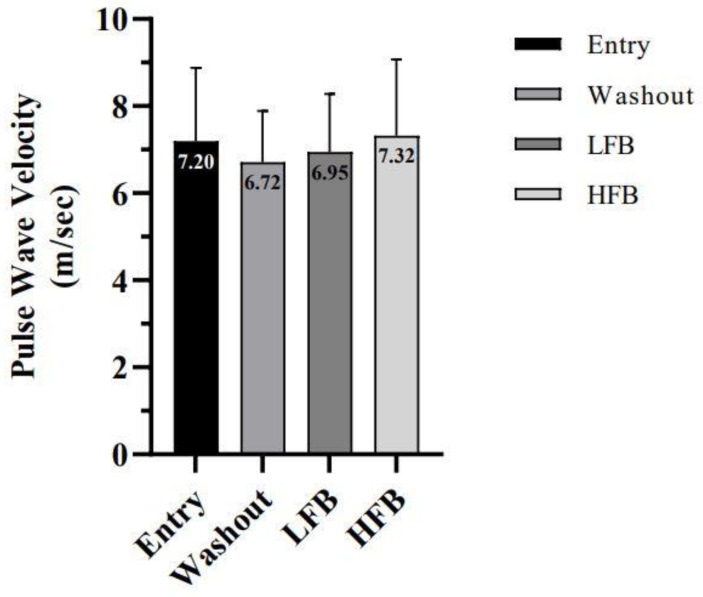
Carotid–femoral pulse wave velocity. Values are for entry, washout, LFB intervention, and HFB intervention. Cross-over design (*n* = 23); values represent mean ± SD. Non-significant: *p* > 0.05.

PWV measurements for each study visit are displayed in Figure 2. There was no significant condition, test, or interaction effect between any study visits.

### 3.3. Resting Blood Pressure

**Figure 3:**
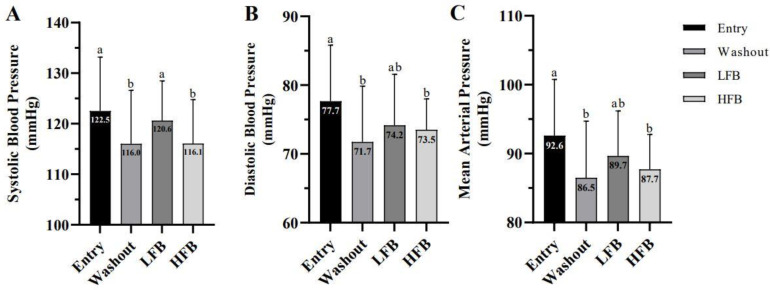
(**A**) Resting systolic blood pressure. (**B**) Resting diastolic blood pressure. (**C**) Mean arterial pressure. Values are for entry, washout, LFB intervention, and HFB intervention. Cross-over design (*n* = 21); values represent mean ± SD. Means without a common letter differ, *p* < 0.05.

Measurements for resting blood pressure are depicted for SBP, DBP, and mean arterial pressure (MAP) in Figure 3A–C. Results revealed a significant condition effect (*p* < 0.01) for SBP. The follow-up paired *t*-test indicated that SBP was lower during the HFB intervention compared to the LFB (*p* = 0.04) and entry visit (*p* < 0.01). Conversely, SBP during the washout was lower than both LFB and entry time points (*p* = 0.02 and 0.01, respectively). No significant differences in SBP existed between the washout and HFB intervention (*p* = 0.80). 

Results for resting DBP revealed a significant condition effect (*p* < 0.01), with the follow-up paired *t*-test indicating that values in the HFB intervention were significantly lower compared to the washout and entry time points (*p* = 0.017 and 0.003, respectively). Similarly, results for MAP revealed a significant condition effect (*p* < 0.01). The paired *t*-test indicated that MAP was significantly lower after the HFB intervention and washout time points relative to entry (*p* = 0.002 for both). No significant condition, test, or interaction effects were demonstrated for resting HR across time points.

### 3.4. Dietary Analysis

The dietary analysis of macronutrients is listed in Table 3. The total caloric intake did not change among any of the study periods. There was a significant test effect (*p* = 0.045) for % energy (% EN) from CHO, with the paired *t*-test indicating that % EN from CHO during the HFB intervention was lower than at entry (*p* = 0.030). Additionally, an interaction effect was seen in % EN from protein and % EN from fat (*p* = 0.011 and 0.034, respectively). % EN from protein and % EN from fat were significantly greater during the LFB and HFB intervention, respectively, than at other time points. Significant interaction effects were also found for daily intakes of total fat, SFAs, and MUFAs (*p* = 0.013, 0.044, and 0.049, respectively) with the simple main effects indicating that intake values were greater during the HFB intervention than at all other time points. 

### 3.5. Aerobic Fitness and Energy Expenditure

There were no significant differences (*p* > 0.05) between the first and last visits for estimated VO_2max_ (entry = 37.92 mL·kg^−1^·min^−1^, final visit = 37.11 mL·kg^−1^·min^−1^), and following washout, LFB, and HFB periods for daily energy expenditure estimated by activity logs (washout = 3656.65 kcal, LFB = 3622.96 kcal, HFB = 3606.65 kcal).

## 4. Discussion

The key findings of this study were that (1) the FMD response was higher after the HFB compared to the LFB intervention; (2) PWV was not different between the HFB and LFB interventions; and (3) SBP and DBP were reduced during the HFB intervention. At entry, participants were below the referenced FMD cutoffs for normal endothelial function [17,18] and remained under either value following the washout and LFB periods, indicating increased CVD risk. Interestingly, the recovery of gated and allometrically scaled FMD during the HFB intervention to the level of normal endothelial function (gated = 8.26%; allometrically scaled = 7.13%) suggests reduced CVD risk. However, there were no significant effects of the ground beef interventions for PWV. In line with previous studies that assessed PWV in response to dietary interventions [24,25], our finding provides additional evidence that PWV is not likely to be altered by short-term dietary interventions. Nevertheless, this study is the first to demonstrate a reduction in BP following short-term high-fat ground beef consumption, a result that runs counter to the widespread conception that high-SFA foods, such as beef, are unhealthy dietary choices.

To our knowledge, this is the first study to assess the vascular outcomes of consuming high-fat vs. low-fat ground beef. Thus, direct comparisons to existing literature are not possible. However, others have examined vascular responses to diets high in fat compared to diets high in CHO [22,23,25] and beef compared to bison [44]. Primarily, diets high in TFAs have detrimental effects on the FMD response, which is unaltered by diets high in CHO [22,23,25]. Additionally, a study that evaluated the vascular effect of bison (8.8–9.5 g fat per serving) compared to ground beef (19.0–21.8 g fat per serving) observed no difference in FMD after a 7-week intervention [44]. 

Contrary to the above findings [44], in the current study, we observed an increased FMD response following 5 weeks of high-fat, high-SFA/TFA ground beef consumption. This increase was observed with no change in total caloric intake, physical fitness, or energy expenditure, suggesting that the increase in FMD occurred independent of these mechanisms. We propose that the improved FMD response is related to the significant decrease in LDL-C reported in Lytle et al. [31], since LDL-C previously has been shown to deleteriously affect the vascular dilatory response by inhibiting NO synthesis and release [45]. Because the HFB intervention also lowered HDL-C [31], a cholesterol subtype that improves vascular function, alterations in HDL functionality potentially affected our FMD results. However, it has been reported that HDL-C concentrations are not nearly as important as HDL functionality, best assessed by apolipoprotein A1 levels [46]. To this end, beef consumption is linked to increased apolipoprotein A1 concentrations in humans [47]. 

Consistent with the findings of other authors [35], the observed increase in MUFA consumption during the HFB intervention may partially explain the increase in FMD. Fuentes et al. [33] compared a Mediterranean-like diet, a low-fat diet, and a high-SFA diet in normocholesterolemic men. The Mediterranean diet, which contained 38% EN from fat with 22% EN from MUFAs, significantly increased the FMD response and decreased LDL-C and TC relative to the low-fat (<28% EN from fat) and high-SFA (38% EN from fat; 20% EN SFAs) diets [33]. Those findings in conjunction with our own indicate that high serum LDL-C attenuates FMD, while short-term diets high in MUFAs can reverse the diminished dilatory response. 

Likewise, the available literature corroborates our observed reduction in BP during the HFB intervention as a result of increased MUFA intake [30,48]. Decreased BP as a result of increased MUFA intake has been reported in both healthy individuals and non-insulin-dependent diabetic individuals [30,48]. The HFB patties contained a much greater amount of MUFAs (primarily oleic acid; 18:1n-9) than the LFB patties, and only the HFB intervention depressed SBP and DBP [31]. Those findings are supported by cross-sectional studies that report an inverse relationship between BP and MUFA consumption [49,50]. Additionally, BP is not increased by high-fat diets enriched with SFAs [29]. It, therefore, appears that in healthy individuals, diets with a larger MUFA content lower BP, whereas diets containing high SFAs do not alter BP [31]. It should be noted that there was a significant carryover effect for SBP, DBP, and MAP, in that these values were significantly lower at washout than at entry. This carryover effect was seen following the LFB intervention; we interpret this to mean that the carryover effect for BP values was the result of the previous consumption of the HFB patties.

This study utilized participants recruited to test the effects of LFB and HFB on lipoprotein cholesterol concentrations and lipoprotein particle abundance [31] and involved 23 of the 25 participants who completed all phases of the lipoprotein cholesterol study. While slightly different in absolute values for nutrient intake, the patterns of % EN and actual nutrient intakes were identical. Thus, consumption of the LFB patties increased protein intake, whereas consumption of the HFB patties increased total fat, saturated fat, and monounsaturated fat intake [31]. Additionally, both LFB and HFB treatments decreased HDL-C and LDL-C concentrations for the 23 participants for the current study, identical to results previously reported [31].

We did not measure the protein content of our ground beef preparations, but the calculated values of protein for LFB and HFB were 23.7 and 18.1 g/113 g patty, respectively (https://fdc.nal.usda.gov/fdc-app.html#/food-details/168652/nutrients, accessed on 12 April 2021). The greater protein content of the LFB was responsible for the increased protein intake during consumption of the LFB patties. As stated above, FMD is a non-invasive measure of NO bioavailability that assesses endothelial function. Arginine, one of the several bioactive components of beef, is a primary substrate for NO synthase [51], and arginine comprises approximately 7% of the total mass of beef proteins [52]. However, neither arginine nor a bioactive peptide in the beef can explain the increase in FMD elicited by the HFB treatment; protein intake did not differ between entry values and following HFB consumption, but only the HFB treatment increased FMD.

Our laboratory has focused on the effects of ground beef fatty acids on lipoprotein cholesterol metabolism [34,36,37,47]. For studies designed to test the effects of dietary nutrients on lipoprotein cholesterol metabolism, a test interval of 5 weeks and a washout period of 4 weeks has been established as adequate to document diet effects and minimize carryover effects, respectively [53]. However, there are insufficient data to establish optimal treatment and washout periods for studies on FMD.

This study is not without limitations. Resulting from a limited staff, participant scheduling was based on availability. While all testing visits took place during the fifth week of the intervention or fourth week of the washout period, the specific day of the week may have been different within and between participants for each intervention. Total funding from the funding agency was sufficient to cover expenses for approximately 30 individuals. Data from previous trials in our laboratory [32,34,36,37] indicated that women had much greater variation in HDL-C concentration at entry (47–120 mg/dL) than men (36–76 mg/dL) (variation in cholesterol concentration not published previously). The range of HDL-C concentrations at entry in the current study (39–73 mg/dL) was similar to the variation in our previous studies with men. The lesser variation in HDL-C concentration for men improved the power of our statistical analyses. Additionally, coronary heart disease mortality is two to five times greater in men than in women, depending on age group [54]. For these reasons, only men were chosen for this trial. Nevertheless, it is unknown whether similar effects of ground beef on FMD can be demonstrated in women. Additionally, while this study utilizes a chronic dietary intervention design, outcomes after only five weeks extrapolated to habitual consumption of the same diet may not be warranted. Lastly, this study does not include the shear rate associated with FMD measures.

## 5. Conclusions

Our results demonstrate that the addition of either low- or high-fat ground beef does not negatively impact vascular function. In fact, the HFB intervention improved the FMD response, which is known to decrease risk of CVD [15,55]. Additional benefits of the HFB intervention included a cardioprotective decrease in both SBP and DBP relative to LFB and entry, and a significant reduction in LDL-C. However, a caveat to the addition of both LFB and HFB to the diet was the reduction in HDL-C. This may or may not be detrimental depending on the functionality of the HDL particles themselves. Future research should investigate HDL functionality to determine whether beef alters these markers, which may be more important than HDL-C concentration alone. Thus, contrary to common conception, our results suggest that HFB may be a healthier choice than LFB when added to the habitual diet.

## Figures and Tables

**Table 1:** Participant demographics at study entry.

	Entry (*n* = 23)
Age (years)	39.9 ± 10.8
Height (cm)	177.5 ± 6.7
Body weight (kg)	97.3 ± 25.0
BMI (kg/m^2^)	31.2 ± 9.0
Lean mass (kg)	64.5 ± 9.5
Fat mass (kg)	30.6 ± 19.1
Body fat (%)	29.9 ± 10.4
Android fat (%)	35.8 ± 14.1
Gynoid fat (%)	31.0 ± 9.8
VO_2max_ (mL/kg/min)	37.9 ± 7.6

Data listed as mean ± standard deviation.

**Table 2:** Baseline and peak brachial artery diameters and time to peak dilation at each study time point.

	Entry	Washout	LFB	HFB
Baseline diameter (mm)	4.45 ± 0.54	4.47 ± 0.60	4.48 ± 0.54	4.45 ± 0.61
Peak diameter (mm)	4.68 ± 0.57	4.73 ± 0.67	4.74 ± 0.58	4.81 ± 0.63
Time to peak dilation (s)	35.1 ± 20.4	32.3 ± 19.7	35.1 ± 22.5	28.4 ± 17.1

Values are for baseline and maximum brachial artery diameter and time to peak dilation at entry, washout, LFB intervention, and HFB intervention. Cross-over design (*n* = 23); values represent mean ± SD. No significant differences were observed, *p* > 0.05.

**Table 3:** Daily macronutrient intake.

	Entry	Washout	LFB	HFB
EN (kcal/day)	2071 ± 491 ^a^	1966 ± 399 ^a^	1887 ± 401 ^a^	2071 ± 487 ^a^
% EN CHO	41.3 ± 8.6 ^a^	41.2 ± 7.4 ^ab^	40.3 ± 8.2 ^ab^	38.3 ± 8.8 ^b^
% EN Protein	18.6 ± 3.5 ^a^	18.9 ± 4.7 ^a^	22.0 ± 4.8 ^b^	18.4 ± 5.8 ^a^
% EN Fat	38.6 ± 6.0 ^a^	37.4 ± 6.6 ^a^	36.8 ± 6.6 ^a^	42.2 ± 8.7 ^b^
Cholesterol (mg/d)	437.2 ± 304.8 ^a^	330.4 ± 179.9 ^a^	344.8 ± 196.7 ^a^	321.7 ± 170.0 ^a^
Protein (g/d)	96.2 ± 28.5 ^a^	91.2 ± 22.8 ^a^	103.9 ± 32.5 ^a^	95.2 ± 38.2 ^a^
CHO (g/d)	212.5 ± 63.9 ^a^	202.4 ± 53.3 ^a^	188.7 ± 48.8 ^a^	197.5 ± 64.0 ^a^
Fat (g/d)	90.0 ± 28.0 ^a^	82.2 ± 24.6 ^a^	76.9 ± 19.1 ^a^	97.1 ± 30.8 ^b^
SFAs (g/d)	29.8 ± 9.5 ^a^	27.9 ± 8.8 ^a^	26.9 ± 8.5 ^a^	34.1 ± 13.0 ^b^
MUFAs (g/d)	15.7 ± 8.5 ^a^	15.3 ± 8.2 ^a^	15.7 ± 7.3 ^a^	22.9 ± 11.2 ^b^
PUFAs (g/d)	8.3 ± 4.3 ^a^	8.2 ± 4.2 ^a^	7.8 ± 4.9 ^a^	7.9 ± 3.6 ^a^
TFAs (g/d)	0.8 ± 0.8 ^a^	0.5 ± 0.5 ^a^	0.3 ± 0.4 ^a^	0.6 ± 0.8 ^a^
n-6 fatty acids (g/d)	6.2 ± 4.0 ^a^	5.7 ± 3.4 ^a^	5.4 ± 4.7 ^a^	5.1 ± 2.7 ^a^
n-3 fatty acids (g/d)	0.7 ± 0.7 ^a^	0.7 ± 0.5 ^a^	0.7 ± 0.6 ^a^	0.5 ± 0.3 ^a^

Values are for the daily intakes of major nutrients of men at entry, washout, LFB intervention, and HFB intervention. Data were derived from 3-day diet records that included 1 weekend day. Cross-over design (*n* = 23); values represent mean ± SD. Means without a common letter differ, *p* < 0.05.

## Data Availability

The data in this study are available upon request from the author.
